# Supplementary material for: The relationship between heart rate variability and glucose clearance in healthy men and women
Source: PLoS One. 2024 Jun 5;19(6):e0303346. doi: 10.1371/journal.pone.0303346 (PMC11152311; doi:10.1371/journal.pone.0303346)
Supplement: Attachment — Submitted filename: PlosOneReviewer.docx [file pone.0303346.s001.docx]

**Reviewer #1:**

The authors have found significant correlations between heart rate variability (HRV) markers and fasting blood glucose levels in healthy individuals. However, they did not find any significant correlations between HRV markers and the area under the curve (AUC) of glucose. When analyzing by sex, only men showed significant correlations between AUC and RMSSD, SDNN, and LF. There were no sex differences for fasting blood glucose levels, AUC, RMSSD, SDNN, HF, and LF.

However, it is important to note that this is a small study with a limited sample size. a sample size of at least 30 is recommended for statistical tests because it is large enough to approximate the true distribution of the population being studied.

The following was changed in the limitations:

“Though this study was a novel attempt to evaluate glucose regulation, it was not without limitations. This study used a small sample size, which is an inherit limitation regardless of significant findings. Future studies should use a more robust sample size to better corollate physical and physiological contributors to HRV based OGTT relationship. Furthermore, this study was not designed to control for menstrual cycle. Future studies should enroll female participants in a fashion to control for stage of menstrual cycle. This study used single-day HRV to predict same-day metabolic function; though appropriate for comparing to same-day OGTT, this value may be stronger when compared to a weekly average, given the levels of variability we observed in these markers. The measurement of glucose concentration in this study was performed using finger-prick, future projects should use vein collection to reduce standard error. Lastly, plasma insulin may provide additional insight for this study.”

1) Was the HRV derived from ECG with your own algorithm, or it was just getting from the ECG monitor?

HRV was derived using Kubios software (version 3.0.5). The files collected from the Finapres NOVA were transferred to Kubios for analysis. The following was edited in the methods to clarify this more.

“Measurements for the current study were collected using the Finapres NOVA, and ECG files were transferred to Kubios software (version 3.0.5).”

2) authors should explain why they chose the statistical measures.

“We used parametric stats appropriate for the nature of our data.   Because parametric stats assume that data are distributed normally, performed a test for normality, to ensure that our data were indeed distributed normally.  We performed two-tailed correlation analysis to be conservative in our conclusions. Likewise, we did a two-tailed independent samples t-test to conservatively compare results between sexes.”

3) The study you provided does not mention the use of a menstrual cycle survey in the correlations of measures, why did the authors performed that survey, the same for the Urine Specific Gravity?

Menstrual cycle information was not entered into the correlation due to the low sample size and was used as observational data. Previous work in our lab suggested a relationship between HRV and menstrual cycle, so we collected the phase data as a participant characteristic in the event we observed a sex-based difference. USG was collected for the BIA hydration

**Reviewer #2:**

The study by Nickel et al. investigated the impact of oral glucose on the extent of HRV, considered an important biomarker for metabolic health. The participants were given a 75-g glucose load, and HRV was measured before its ingestion. Different HRV calculations/parameters were used to determine its association with the OGTT response (assessed as the AUC). The results showed that HRV parameters were correlated with capillary glucose concentration. Considering sex in the statistical model, correlations disappeared in women but not in men. Furthermore, HRV and AUC parameters were also correlated in the group of men. The research was properly conducted; methods are appropriate, and results might have certain implications regarding the HRV field. I have the following constructive comments that hopefully can further increase the quality of this manuscript.

Method section:
The study was carefully designed and conducted. However, the sample size was small (n=18; 10 men and 8 women). Could authors mention this issue (I missed this information in the Method section)? As the small sample size included in the study might impact data interpretation. Thank you.

This information we added to the limitation section.

Could the authors better explain what “apparently healthy individuals” is? Did authors determine that using blood samples, personal/medical interviews, questionnaires or is self-reported? Please provide more information as the paper is focused on “healthy individuals.”

“Inclusion and exclusion criteria were determined through self-report through the health history questionnaire”

Was glucose metabolism impairment established as an inclusion/exclusion criterion? If yes, how was it determined?

Known metabolic conditions were a part of the exclusion criteria and determined through the self-report HHQ.

Regarding glucose concentration, authors should mention they are measuring “capillary blood glucose” instead of “blood glucose.”

Fixed

Could authors provide the exact fasting time (e.g., mean and SD). Thank you.
We only confirmed that they had not eaten prior to 12 hours before arriving to the lab, so we do not have exact times.
Was a commercial glucose drink used, or "homemade" - please state the source and volume (this information was not provided).

OGTT beverage containing 75 grams of glucose (Trutol^TM^)

Does the participant have an “acclimation period” prior to the HRV assessment, or did the HRV assessment start immediately after participants were placed in bed/stretcher?

The following was added:

“on an examination bed” was added to the Experimental Design section.

“The first five minutes of the 10-minute recording were discarded for acclimatation. The last five minutes of resting time points were analyzed.”

I noticed authors used the low artifact correction filter in the Kubios software. Could the authors better explain why they used this filter? In a relatively recent study (PMID: 31979367), the authors proposed the medium filter for young adults. In this regard, did the authors check whether the results are independent of the Kubios filter used?

We visually inspected each recording for artifact when applying the filters and used the lowest filter setting possible to avoid augmenting the recording beyond the natural variations that may occur. The citations we used warned against too strong of filters as it may “over filter” the recording and lose natural variation. The following was added for clarification:

The Kubios “low artifact correction” filter with a ± 0.35 sec sensitivity to R-R abnormalities compared to the local average was used [12,13].

AUC calculation: I understood the rationale for computing the AUC using the Tai’s Mathematical Model; however, I wonder whether that AUC calculation provides glucose values as “arbitrary units” or “no units” (as certain AUC calculations [e.g., PMID: 14756916]). Could authors mention this aspect?

The reviewer brings up a good point, the unit used in this model was “arbitrary units”, this has been added into the manuscript and is expressed as AU.

The thermic effect of a 75g bolus of glucose was unlikely to have been completed within the 3-hour measurement window - therefore it is possible that some differences in the response may have been missed by not capturing the downward tail of the HRV response curve. Should this not warrant a mention?

The reviewer makes a good point. The duration of the OGTT used was the standard two hours. However, we added the following to the limitation section:

Future studies should extend the number of measures glucose time points beyond the two-hour mark in order to capture the downward tail of the OGTT and capture full glucose clearance.

Moreover, did capillary glucose concentration values return to baseline? If not, do the authors believe this issue could partially explain the results (e.g., absence of associations)? If capillary glucose did not return to baseline levels after 2-h, glucose-stimulation could be influencing the results. I would suggest mention this issue.

Capillary glucose concentration values did not return to baseline (Figure 4). We believe this was addressed with the change we made with the previous comment.

Finally, could authors provide a figure showing the capillary glucose concentration and across time (i.e., the curve)? Thank you.

Figure 4 was added for capillary glucose over time

Does a protocol of repeated finger-prick collection add to within-subject variability? Capillary blood is notoriously difficult to standardize at each time point of collection, though it may relate to venous values.

This is a good comment and we added the limitation of finger sticks with future work utilizing venipuncture.

Lines 133-135: No information regarding missing data is provided. Although Table 2 included 18 participants, I noticed that Figures (1 – 3) showed 15 participants instead of 18. Please mention this issue.

Three participants were dropped due to ECG error, they were removed from analysis and we accidentally left the total group in the table. Table 2 has been updated and the following was added:

“Eighteen apparently healthy volunteers completed the study: ten males and eight females. Two males and one female were removed from data analysis due to artifact or ECG recording error, 15 total participants were analyzed, eight males and seven females.”

Why was the menstrual cycle recorded/registered if it was not used for analyses? Did the authors include the menstrual cycle in the analyses (e.g., as a confounder factor for correlations)? If not, I would suggest placing Table 1 as supplementary material or embed it in Table 2.

Menstrual cycle information was not entered into the correlation due to the low sample size and was used as observational data. Previous work in our lab suggested a relationship between HRV and menstrual cycle, so we collected the phase data as a participant characteristic in the event we observed a sex-based difference.

New analysis: I would suggest repeating the analyses, including heart rate (in beats per minute).

RHR correlation data was added to table 3 and results.

Discussion section:

Lines 173-175: This could be related to the low statistical power/sample size, rather than not an effect was observed. This issue deserves a mention.

Sample size was added as a limitation to the limitation section.

Lines 179-181: This threshold is commonly used when blood glucose concentration (i.e., vein samples) is obtained. As I mentioned previously, I would recommend emphasizing that capillary instead of vein glucose concentration was assessed.

The following was edited:

“The participants in this study were considered healthy, normal weight individuals with an average capillary FBG of 89.9 ± 8.2 mg/dL.”

Limitation section:

In my opinion, the following should be included: The small sample size should be mentioned in this section. The menstrual cycle should be mentioned as a potential limitation, as women were at different menstrual cycle statuses which could impact the results in an unknown manner. Glucose concentration was assessed by a finger-prick instead of vein collection.

The following was changed in the limitations:

“Though this study was a novel attempt to evaluate glucose regulation, it was not without limitations. This study used a small sample size, which is an inherit limitation regardless of significant findings. Future studies should use a more robust sample size to better corollate physical and physiological contributors to HRV based OGTT relationship. Furthermore, this study was not designed to control for menstrual cycle. Future studies should enroll female participants in a fashion to control for stage of menstrual cycle. This study used single-day HRV to predict same-day metabolic function; though appropriate for comparing to same-day OGTT, this value may be stronger when compared to a weekly average, given the levels of variability we observed in these markers. The measurement of glucose concentration in this study was performed using finger-prick, future projects should use vein collection to reduce standard error. Lastly, plasma insulin may provide additional insight for this study.”
