# Supplementary material for: The relationship between heart rate variability and glucose clearance in healthy men and women
Source: PLoS One. 2024 Jun 5;19(6):e0303346. doi: 10.1371/journal.pone.0303346 (PMC11152311; doi:10.1371/journal.pone.0303346)
Supplement: Attachment — Submitted filename: Reviewer comments 2.docx [file pone.0303346.s002.docx]

The authors would like to thank the reviewer for their time and recommendations for the manuscript. Responses and changes can be found below.

Reviewer #2: I would like to thank the authors for taking the time to make revisions based on my comments and suggestions. Please find below my minor comments and suggestions:

1. I would suggest adding the lack of an exact fasting time to the limitations section, as the authors 'recommended' fasting for ≥ 12 hours, but cannot confirm it.

We re-examined the data files and found the following:

Participant fasting times were; Total - 13.1 +/- 1.8 hours, Males - 12.6 +/- 2.1 hours, and Females - 13.7 +/- 1.2 hours.

2. References 12 and 13 are cited to justify Kubios artifacts 'over filter' correction. I would suggest including the following citation (PMID: 31979367) as it addresses this issue.

The following citation was added:

Alcantara JMA, Plaza-Florido A, Amaro-Gahete FJ, Acosta FM, Migueles JH, Molina-Garcia P, et al. Impact of Using Different Levels of Threshold-Based Artefact Correction on the Quantification of Heart Rate Variability in Three Independent Human Cohorts. J Clin Med. 2020 Jan 23;9(2):325.

3. How resting heart rate (RHR) was derived from the ECG signal should be added to the methods section. Additionally, please check the units of RHR in Table 3 (should be bpm instead of bmp).

The following was changed:

Methods: HRV Analysis section

The last five minutes of resting time points were analyzed for HRV and RHR.

Bmp was changed to bpm.

4. I noticed that information concerning detrending, lambda, and interpolation rate was not provided in the Kubios software description.

Detrending was set to smoothing priors with a smoothing parameter of 500, lambda of 0.035 Hz, and Interpolation rate of 4 Hz

5. In the Methods section, when referring to the Fast Fourier Transformation technique, I would suggest changing 'technique' to 'algorithm' for clarity.

This change was made
